# Estimation of Continuous Blood Pressure from PPG via a Federated Learning Approach

**DOI:** 10.3390/s21186311

**Published:** 2021-09-21

**Authors:** Eoin Brophy, Maarten De Vos, Geraldine Boylan, Tomás Ward

**Affiliations:** 1 Infant Research Centre, University College Cork, Cork T12 YN60, Ireland; g.boylan@ucc.ie; 2 School of Computing, Dublin City University, Dublin 9, Ireland; tomas.ward@dcu.ie; 3 Department of Electrical Engineering, KU Leuven, 3000 Leuven, Belgium; maarten.devos@kuleuven.be; 4 Insight SFI Research Centre for Data Analytics, Dublin City University, Dublin 9, Ireland

**Keywords:** GAN, blood pressure, photoplethysmogram, time series

## Abstract

Ischemic heart disease is the highest cause of mortality globally each year. This puts a massive strain not only on the lives of those affected, but also on the public healthcare systems. To understand the dynamics of the healthy and unhealthy heart, doctors commonly use an electrocardiogram (ECG) and blood pressure (BP) readings. These methods are often quite invasive, particularly when continuous arterial blood pressure (ABP) readings are taken, and not to mention very costly. Using machine learning methods, we develop a framework capable of inferring ABP from a single optical photoplethysmogram (PPG) sensor alone. We train our framework across distributed models and data sources to mimic a large-scale distributed collaborative learning experiment that could be implemented across low-cost wearables. Our time-series-to-time-series generative adversarial network (T2TGAN) is capable of high-quality continuous ABP generation from a PPG signal with a mean error of 2.95 mmHg and a standard deviation of 19.33 mmHg when estimating mean arterial pressure on a previously unseen, noisy, independent dataset. To our knowledge, this framework is the first example of a GAN capable of continuous ABP generation from an input PPG signal that also uses a federated learning methodology.

## 1. Introduction

Chronic heart disease was the number one cause of death from 2000 to 2019, according to the World Health Organisation (WHO), and was responsible for 16% of the total worldwide deaths in 2019 [1]. Heart disease has also shown the most significant increase in deaths during this period. Obtaining unobtrusive, continuous measurements of the cardiac state has proven very difficult. The most commonly used indicator for measuring the state of the heart is blood pressure (BP), which is often gathered using a sphygmomanometer cuff, a finapres, or an arterial catheter. Sphygmomanometers provide spot measurements for BP over a very short time interval, and arterial catheters are an extremely invasive method of continuous BP measurement. The finapres is an alternative for continuous and unobtrusive BP measurement. However, these devices’ size, shape, and price mean that they have not been commoditised for individuals seeking continuous home BP measurement devices. Regular monitoring of BP can prove vital for people suffering from cardiovascular diseases (CVDs) who are already vulnerable to BP fluctuations.

Methods for non-invasively measuring continuous arterial blood pressure (ABP) have been explored, using other physiological signals to infer ABP. One example uses the pulse transit time (PTT), the time interval taken for a pulse wave to travel between two arterial sites. PTT varies inversely to BP changes and has been demonstrated to be a valid and accepted measure of BP [2,3]. PTT is formally defined as the time interval between the Q wave of the electrocardiogram (ECG) signal and the pulse’s arrival at a peripheral site. As such, this information should also be available from a photoplethysmography (PPG) signal.

PPG is an optical technique that requires a single sensor and has become commoditised in the past number of years such that it is included in most wearables and other medical devices. It works by shining a light-emitting diode (LED) into the microvascular tissue, measuring the amount of light reflected/transmitted/absorbed via a photo-sensor, and detecting blood volume changes over the cardiac cycle. The output from this sensor is then conditioned so a valid heart rate can be determined. Furthermore, having a continuous heart rate measurement means we can extract a PTT measurement, which means that providing further analysis, we can extract meaningful ABP measurements using a PPG sensor alone.

Our work described here is part of a larger-scoped effort to develop readily deployable artificial intelligence (AI) systems that non-expert consumers and downstream end-users can easily interpret. Capitalising on recent advancements in machine learning has the potential to simplify wearable devices, allowing for a reduction in power requirements and, subsequently, lower-cost devices, as our previous work also aims to achieve [4].

This paper presents our novel framework for implementing continuous ABP measurement using a PPG sensor alone. Our methods use proven cutting-edge machine-learning techniques to capture the characteristics that correlate and link continuous PPG to continuous ABP measurements. For the first time, we demonstrate a decentralised learning approach to continuous ABP measurement that is capable of real-world implementation on a large scale and does not compromise patient privacy. This novel approach yields a more power-efficient learning framework, thus advancing the development of simpler, more cost-effective wearables without compromising the accuracy of ABP measurements and patient privacy.

## 2. Related Works

Many works in the past have focused on estimating BP from correlating features available in ECG, and PPG [2,3,5,6]. These works have demonstrated high-quality results in ABP estimation but require domain expertise to process the available PPG signal to acquire the blood pressure estimation. Many machine-learning methods are being developed to remove the dependency on signal processing experts, allowing for readily deployable AI systems. Our framework follows suit in automating the signal extraction process, making handcrafted feature selection obsolete.

Slapničar et al. implemented a spectro-temporal deep neural network (DNN) to model the dependencies that exist between PPG and BP waveforms. The authors used a PPG signal along with its first and second derivatives and determined the network that is successful at modelling the dependent characteristics of BP [7]. El Hajj and Kyriacou implemented recurrent neural networks (RNNs) for the estimation of BP from PPG only [8]. Other works develop a statistical feature extraction and selection process followed by a regression-based predictive model, all of which achieve high-quality BP estimation results from PPG data only [9]. Feature-free methods of BP estimation have also been completed previously through the use of deep-learning-based prediction techniques with good results [10,11]. However, these methods discussed thus far deal with BP prediction in discrete intervals, and we build on this through the generation of continuous BP waveforms and BP prediction.

Ibtehaz and Rahman introduced another feature-free deep-learning method of non-invasive continuous blood pressure modelling [12]. The authors presented their PPG2ABP method that utilises a deep-supervised U-Net model that consists of an encoder and decoder network adopted for regression. In this configuration, their model can predict a complete continuous BP waveform from a PPG waveform. To build on these works, our framework implements a long short-term memory (LSTM) convolutional neural network (CNN) (LSTM-CNN) GAN model that is capable of generating continuous BP from a given PPG signal. Not only is our model capable of PPG2ABP, but also ABP2PPG; this enables our model to infer one physiological time-series waveform from another. Our model can map any given time series signal to another, but for the transform to make sense, the signals should be correlated somehow.

Smartwatches have become pervasive in recent years, but are still technically lacking in terms of sensors available to the end-users. Ion et al. presents a wearable pressure sensor for blood pressure monitoring [13]. Their proposed pressure sensor has the potential for low production costs and integration into wearable devices, yet this technology needs time to mature until it reaches a pervasive computing status. Mena et al. developed a mobile system for non-invasive, continuous BP monitoring [14]. Their system actively collected and transmitted PPG readings from a wrist-mounted sensor to a smartphone, where BP estimation is computed with machine-learning algorithms. Our framework complements and builds on these approaches in employing a decentralised multi-user learning framework enabling more accurate predictive models and, in turn, faster patient diagnoses.

PPG has become a staple sensor in wearables and the primary means of measuring the heart rate of end-users. Yet, from a medical perspective, ECG is the proven and more information-rich signal to measure the cardiac state. In addressing this issue, Sarkar and Etemad [15] present their model CardioGAN that employs an adversarial training method to map PPG to ECG signals. CardioGAN utilises both time- and frequency-domain features of the PPG to generate reliable 4-s long ECG signals. Our approach implements a time-domain-only discriminator to reduce an individual model’s overhead and is capable of generating 10-s long PPG and ABP waveforms from one another. We also take into account the personal data-preserving methods and demonstrate real-world applications of such models.

Addressing data sharing and privacy issues, we adopt a federated learning approach that was initially introduced by Google’s AI Blog [16] as a means to collaborate machine learning across mobile devices without the need to store data in a centralised repository. It enables remote devices to learn collaboratively with a shared global model while keeping their training data on individual devices. The individual client models train locally and send their weights to be aggregated on the global model, which can then be passed back as updated training weights for the client models where training can continue. This entire process is known as a communication round. Rasouli et al. [17] presented one of the first examples of implementing this training process as part of a GAN on image and energy data. We implement a similar training strategy for our framework to serve as proof of concept for distributed training across smartwatches to build a model such as the one presented in our paper.

## 3. Materials and Methods

**Figure 1:**
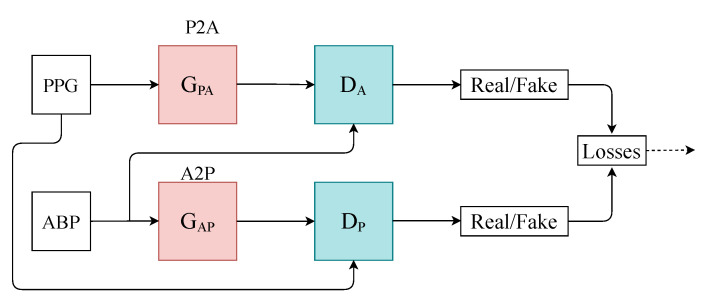
Architecture of the T2T-GAN. P2A represents the generator transform function from PPG to ABP. Conversely, A2P represents ABP to PPG.

We designed a time-series-to-time-series generative adversarial network (T2T-GAN) (Figure 1) based on the popular CycleGAN that is capable of unpaired image-to-image translation [18]. The T2T-GAN can translate from one time-series modality to another using cycle-consistency losses. More specifically, we implemented the T2T-GAN for capturing the complex characteristic relationship between ABP and PPG and trained this model to translate a PPG measurement into an accurate continuous ABP measurement. We opted for a decentralised learning approach here and implement federated learning in the interest of data privacy and protection and real-world implementation. With one central aggregate model and many decentralised models, we can implement our framework without handling individuals’ personal sensitive data. Comprehensive details of our method can be found in the section that follows.

### 3.1. Computing Platform

The experiments for this project were run on an Nvidia Titan Xp with PyTorch and Google Colaboratory in the interests of making the project readily deployable. The code for these experiments is available online (GitHub Repository: https://github.com/Brophy-E/T2TGAN. Date last accessed: 13 September 2021).

### 3.2. Datasets

Two open-source datasets were used in this experiment. The first dataset, “Cuff-Less Blood Pressure Estimation”, is freely available on Kaggle and UCI Machine Learning Repository. It contains preprocessed and validated ECG (electrocardiograms from channel II), PPG (fingertip) and ABP (invasive arterial blood pressure (mmHg)) signals all sampled at 125 Hz [19,20]. The raw ECG, PPG, and ABP signals were originally collected from PhysioNet [21]. This dataset is split into multiple parts and consists of several records; for our work, we used the first 5 (part1.mat–part5.mat) records and segmented them into 8-s intervals, which yielded 144,000 training samples (320 h), and the last 2 (part11.mat–part12.mat) records into 55,000 validation samples (122 h). However, as there might be more than one record per patient (which is not possible to distinguish), we use a second unrelated dataset to test our framework and observe its generalisability. Therefore, we used a [144,000, 2, 1000] dimensional vector that constituted the training dataset for our framework.

**Figure 2:**
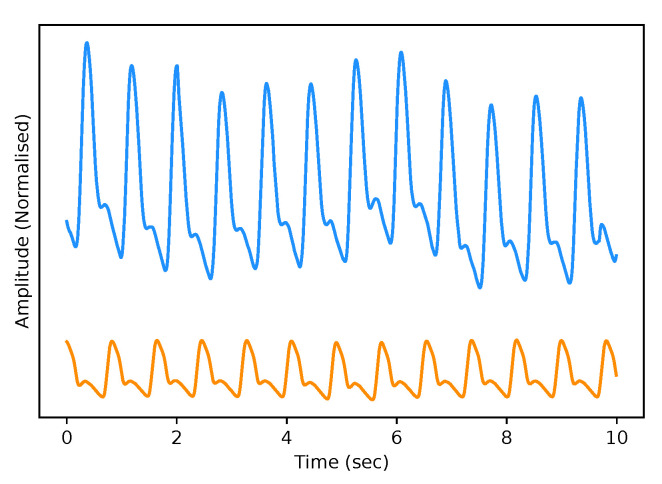
Example of Real PPG (top, blue) and ABP (bottom, orange). The signals are both normalised between 0 and 1 with an artificial offset on the ABP signal for visualisation purposes.

The test dataset “University of Queensland vital signs dataset: development of an accessible repository of anaesthesia patient monitoring data for research” [22] provides a multitude of vital sign waveform data recorded from patients undergoing anaesthesia at the Royal Adelaide Hospital. The physical state of patients under anaesthesia contains marked changes to cardiovascular variables compared to ICU patients, presenting a further challenge to our framework. We are only concerned with the ABP and PPG measurements from this dataset; these are sampled at 100 Hz. We selected only one patient, namely Case 5, and segmented the data into 10-s intervals, which yields a [900, 2, 1000] dimensional vector (150 min) that constitutes the test dataset for our framework. We are only concerned with the PPG and ABP signals from these datasets; see Figure 2 for an example of the real data used in this work.

### 3.3. Model

**Figure 3:**
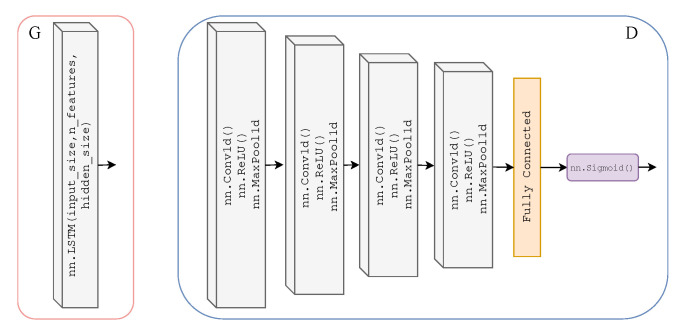
Architecture of Generators $G_{PA}$ and $G_{AP}$ (**left**) which are two-layer stacked LSTMs with 50 hidden units in each layer and a fully connected layer at the output, with an input size of 1000. Architecture of Discriminators $D_{A}$ and $D_{P}$ (**right**) which are 4-layer 1-dimensional CNNs (ReLU activation and max pooling functions) with a fully connected layer and sigmoid activation function at the output.

As previously mentioned, we adopted the learning framework of CycleGAN for time-series data to translate from one time-series modality to another. Here, we will explicitly define the Discriminator and Generator architecture of our T2T-GAN. The Generators $G_{PA}$ and $G_{AP}$ are two-layer stacked LSTMs with 50 hidden units in each layer and a fully connected layer at the output, with an input size of 1000; see Figure 3. The Discriminators $D_{A}$ and $D_{P}$ are 4-layer 1-dimensional CNN with a fully connected layer and sigmoid activation function at the output; see Figure 3.

### 3.4. Federated Learning

**Figure 4:**
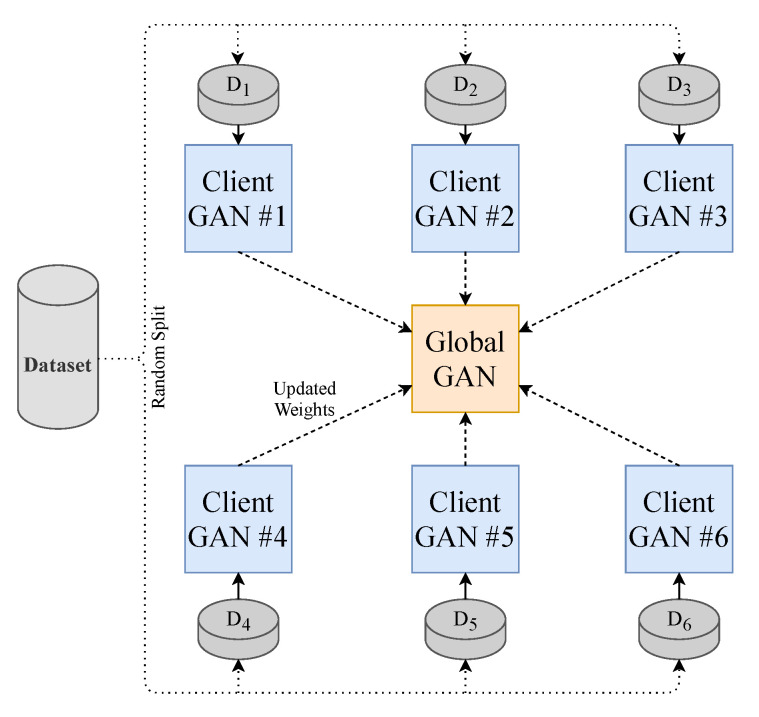
Federated Learning methodology employed in this paper. Each GAN is represented by the model shown previously in Figure 1.

**Figure 5:**
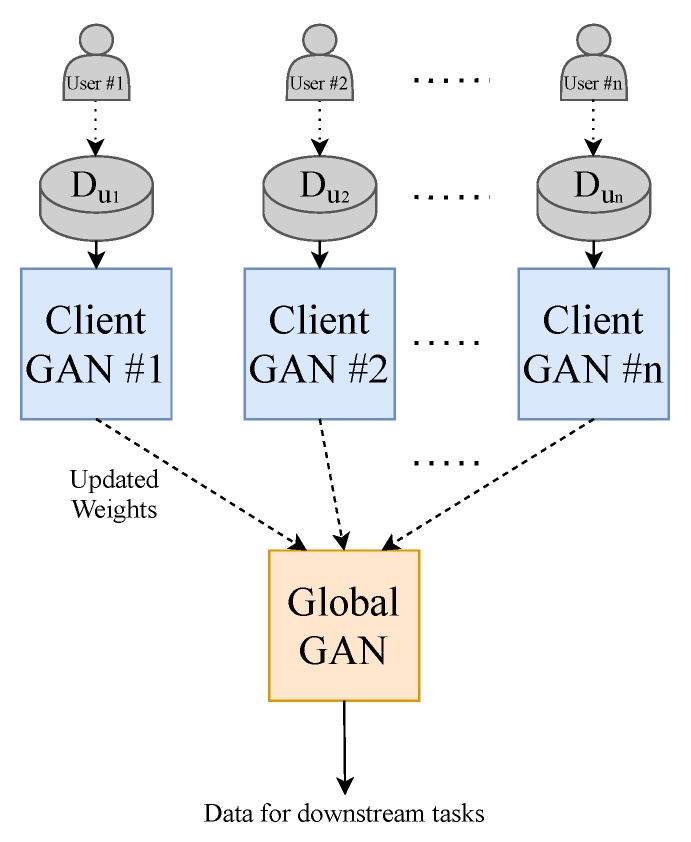
Federated Learning methodology that is implemented in the real world. Each GAN is represented by the model shown previously in Figure 1.

To make the model perform closer to a real-world setting and to prevent data sharing to third parties, we implement the decentralised learning approach of Federated Learning. Our approach is limited to using one central server. To realise this learning method, we split our dataset into *N* (where N=20) equally sized random smaller data subsets and train *N* client-GANs on their own data with no cross-over from their respective subsets. The client-GANs are trained until convergence for *e* (where e=5) epochs, and their weights are then sent to a global-GAN that aggregates the received weights from the *N* clients-GANs. This global-GAN can then operate on unseen data or update the client-GANs with the aggregated global weights, which eliminate the need for any data centralisation; see Figure 4 for a visual example of our method. Of course, in a real-world training and testing environment, the training data will not come from a centralised repository. The end-users will instead generate the data. Consumers will generate their own PPG data from their smartwatch, in this case, that will be used to train a local model and communicate weights to and from a global model, see Figure 5 for a conceptual example.

### 3.5. Training

We chose a total of 20 client models for training as demonstrated in Figure 4. Each used an equal proportion of the dataset. Six random clients were selected from the total client models in each communication round to be trained. There were ten communication rounds. Following each round of training on the client device, the aggregation of weights is computed on the global model. The total number of training rounds on each client was 5, with a batch size of 32. The total loss function of our T2T-GAN framework is calculated as in Equation (1).

(1) $$\begin{array}{c} L\left(P2A,A2P,D_{P},D_{A}\right)=L_{T2T-GAN}\left(P2A,D_{A},PPG,ABP\right)\\ \;+L_{T2T-GAN}\left(A2P,D_{P},ABP,PPG\right) \end{array}$$

where $L_{cyc}$ and $L_{identity}$ are the cycle consistency loss and identity loss, respectively, and are defined by the L1-norm. $L_{T2T-GAN}$ is defined as the mean squared error loss (MSE). λ controls the relative importance of the two objectives, $\lambda_{c}$ and $\lambda_{i}$ were chosen as 10 and 5, respectively, as we want to emphasise the importance of cycling between the time series modalities (PPG to ABP). See Algorithm 1 for a full description of the training procedure.
**Algorithm 1** FedT2TGAN Training Procedure.**Input:** Training sample pairs of ABP and PPG $S_{n}$ = ($X_{ABP1,PPG1}+\ldots +X_{ABPn,PPGn}$)**Output:** GAN Model T2TGANInitialise global-modelSynchronise client-models with global-model**for** num clients **do**    # *Communication Round*    Select 6 random client-models to train    **for** each client-model **do**        # *client-model training*        **for** num epochs **do**           # *Calculate identity, cycle and adversarial losses*           *L* = GetGANLosses($X_{ABP,PPG}$, ${\hat{X}}_{\hat{ABP},\hat{PPG}}$)           # *Update weights of client-model*           *W* = UpdateClientWeights(L)        **end for**        Aggregate client-model’s weights with global-model    **end for****end for**Generate ABP waveforms from unseen PPG using trained global-model

### 3.6. Evaluation

To successfully evaluate our model, we examine the mean arterial pressure (MAP) of generated samples. Using a completely independent test dataset from the training dataset grants us the freedom to implement a leave-one-out strategy and see how well our model generalises to other ABP-PPG datasets. We take the PPG measurements from the test dataset and pass them through our trained global deterministic function, P2A. This function converts our PPG signal to a corresponding ABP signal, and we then calculate the MAP from the generated signal and compare it with the true MAP measurements from the real ABP signal. MAP is considered a better indicator of perfusion to vital organs than systolic blood pressure (SBP) [23]. It is important to note that we can retrieve the systolic and diastolic blood pressure (DBP) from the P2A signal, which we use to calculate the MAP (2) rather than simply returning the mean of the continuous signal segment. We also present the Bland–Altman (BA) plots of the MAP error [24] that allow us to determine to what degree the generated ABP is a good substitute for the real ABP. The Association for the Advancement of Medical Instrumentation (AAMI) standard requires BP measuring methods to have a mean error (μ) and standard deviation (σ) of less than 5 mmHg and 8 mmHg, respectively, [25]. Following this, we then select the entire 150-min period of the test data and perform calibration on these data for the first one-minute period only. This calibration is designed to remove user bias and provide more accurate results while mimicking a continuous BP measurement test that can be performed clinically. Bland–Altman plots are provided for the calibrated and uncalibrated measurements.

(2) MAP=[SBP+(2∗DBP)]/3

In the interest of providing a comprehensive evaluation of our T2TGAN, we implement the dynamic time warping (DTW), root-mean-squared error (RMSE) and Pearson Correlation Coefficient (PCC) algorithms as distance and similarity measures between the real and generated time series BP sequences for both the federated and un-federated approaches. These metrics allow us to quantify the similarities in the structure of the blood pressure waveforms. This is implemented for the entire test dataset (150 min, 900 samples at 10 s/sample) and a random sample of the validation dataset of equal size.

## 4. Results

**Figure 6:**
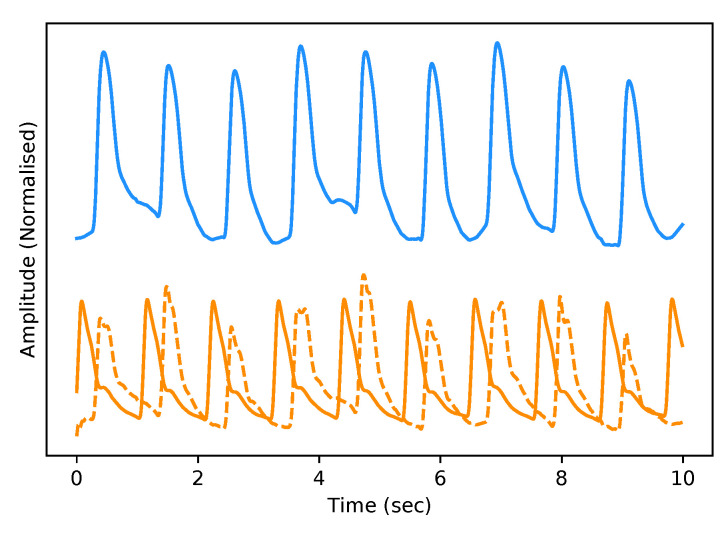
Example of Real PPG (top, blue) and the corresponding real ABP (bottom, dashed-orange) along with the fake ABP (bottom, orange) generated using the respective PPG. The signals are both normalised between 0 and 1 with an artificial offset on the ABP signals for visualisation purposes.

As stated previously in Section 3.6, we evaluate our framework based on a qualitative and quantitative perspective. Visually, and therefore from a subjective qualitative perspective, we determine that our federated T2TGAN framework has successfully modelled ABP from a single optical PPG signal alone. An example of real and generated data can be seen in Figure 6 below.

**Figure 7:**
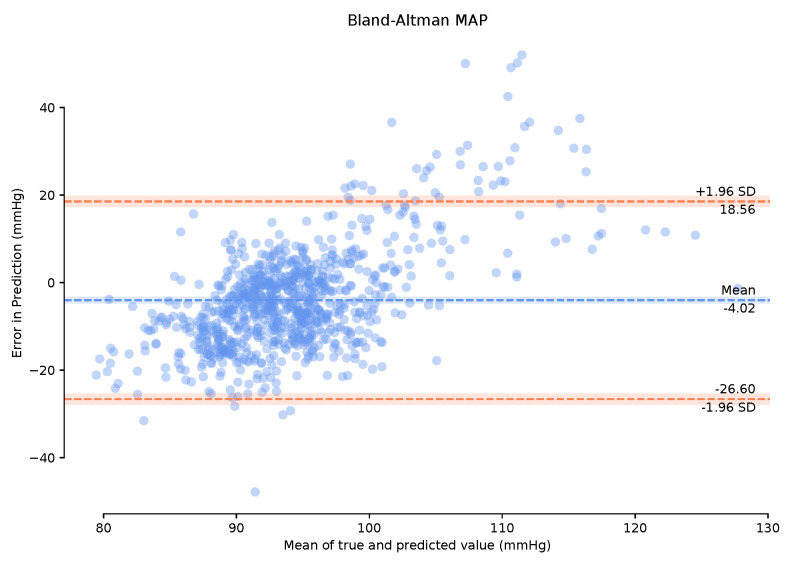
Bland–Altman plots of Mean Arterial Pressure on the unseen, unprocessed test data with a mean error of −4.02 mmHg standard deviation of 22.6 mmHg.

**Figure 8:**
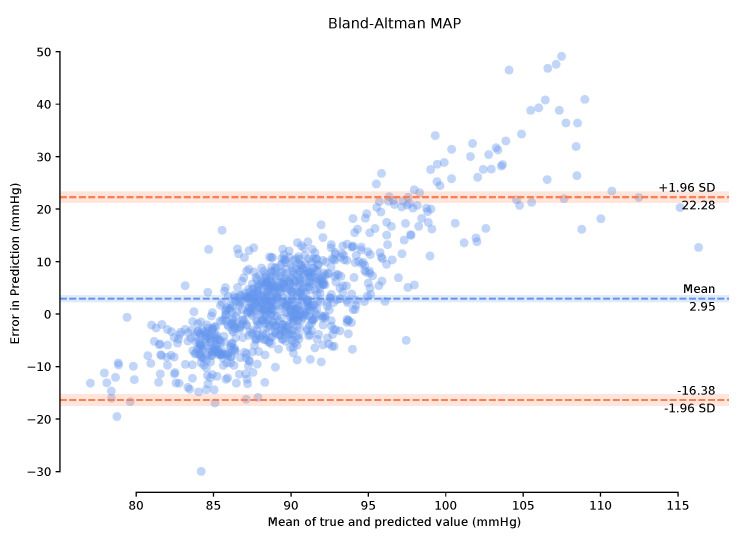
Bland–Altman plots of Mean Arterial Pressure on the unseen, unprocessed test data following a one-minute calibration period with a mean error of 2.95 mmHg standard deviation of 19.33 mmHg.

Observing the Bland–Altman plot in Figure 7 our framework achieved a mean MAP error of −4.02 mmHg and a standard deviation of 22.6 mmHg. We also present the Bland–Altman plots over the 149-min period with a 1-min calibration period that achieved a mean MAP error of 2.95 mmHg and a standard deviation of 19.33 mmHg. This calibration period can prove useful in bringing the mean error to within the AAMI standards. The BA plots show the 95% range from μ−1.96σ to μ+1.96σ. The MAP range of [25.16 mmHg, −20.08 mmHg] in Figure 8 demonstrates that the one-minute calibration period was successful in reducing the overall bias in the mean error.

However, a qualitative evaluation cannot be considered a successful framework justification due to the lack of a suitable objective measure. Therefore, we compute DTW, RMSE error, and PCC of the real vs. generated continuous ABP signals from a quantitative perspective. The time-series similarity results on the validation and test datasets for both the federated and un-federated frameworks are displayed in Table 1 below. It can be seen that, as expected, the federated results are degraded slightly compared to the non-federated results. However, in both cases, the models perform seemly equal on the validation dataset as they do on the test dataset.

**Figure 9:**
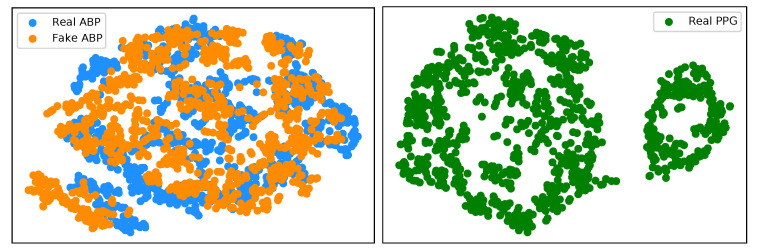
(**left**) t-SNE visualisation of real ABP (blue) and generated ABP (orange) dataset. (**right**) t-SNE visualisation of the real PPG dataset.

We have implemented an explainable AI (XAI) approach known as t-Distributed Stochastic Neighbor Embedding (t-SNE) [26] in the interest of trustworthy AI. This is a well-known technique of dimensionality reduction that is suited well to the visualisation of high-dimensional datasets. We implement t-SNE on our real ABP and PPG test datasets, as well as the generated ABP data. Figure 9 (left) illustrates a clustering effect between the real and synthetically generated ABP that is distinctly different from the t-SNE embedding on the PPG data. This demonstrates that we can now effectively cycle the time series PPG data from its own modality (Figure 9 (right)) to a distribution that is much more representative of the ABP data.

## 5. Discussion and Conclusions

Here, we have presented a novel decentralised learning framework for generating continuous ABP data and MAP estimates using a single optical sensor alone. Although our results of a mean error of 2.95 mmHg and a standard deviation of 19.33 mmHg do not meet the AAMI criterion, it must be stated that for our test dataset, we obtained a completely separate dataset and carried out no further processing on the retrieved data other than segmentation. Our framework performs deceptively well due to the real-world nature of the test dataset and the fact that, as stated before, the physical state of patients under anaesthesia contains marked changes in their cardiovascular variables (ABP and PPG in this case) in comparison to patients in the ICU (training dataset). With further work on cleaning and preprocessing the datasets, we might observe improved results, such as the results observed in [12]. However, we did not implement this as part of this work in keeping with noisy real-world data. In the case of using denoising methods for PPG signals to obtain clean training data, we can seek to apply the techniques listed in [27]. Furthermore, with the increasing environmental costs of machine-learning practises worldwide, we are concerned with model complexity and training time. Our model takes a total time of 5 h to train compared to the 11–12 days to train the models in [12]. We also add a layer of interpretability to our results through the use of t-SNE, which demonstrates that the T2TGAN can successfully cycle one time-series modality to another. Although this work did not achieve the competitive performance of fully connected networks, it should be noted that this work is conceptually quite different from more conventional approaches and opens up new opportunities for consideration, particularly regarding federated learning and privacy.

Sustainable AI is an essential practice in the research community to continuously build quality machine learning systems while consuming smaller amounts of power. Furthermore, explainable AI is crucial to bridge the gap between human and computer understanding and build human trust in these AI systems. Overall our framework lays the foundation for continuous ABP measurements on a large scale for the first time by providing a sustainable, explainable, and private real-world example of how our models learn from small subsets of personal data and generalise well to previously unseen data. Achieving all this while using a sole PPG sensor will subsequently lead to lower-cost devices and the commoditisation of such. This may be one such solution for clinicians to remotely and accurately monitor patients’ cardiovascular states in their fight against CVDs.

## Figures and Tables

**Table 1:** Time series similarity metrics.

Federated Learning	Dataset	DTW	RMSE	PCC
No	Test Dataset	56.73	0.19	−0.11
No	Validation Dataset	55.18	0.23	−0.33
Yes	Test Dataset	62.55	0.24	−0.22
Yes	Validation Dataset	62.15	0.25	−0.34

## Data Availability

Two open-source datasets were used in this experiment. The first dataset “Cuff-Less Blood Pressure Estimation” is freely available on both Kaggle and UCI Machine Learning Repository https://www.kaggle.com/mkachuee/BloodPressureDataset. Date last accessed: 1 September 2021. The second dataset “University of Queensland vital signs dataset: development of an accessible repository of anesthesia patient monitoring data for research” is available at http://journals.lww.com/00000539-201203000-00015. Date last accessed: 1 September 2021.
